# Cystatin C and creatinine-based eGFR levels and their correlation to long-term morbidity and mortality in older adults

**DOI:** 10.1007/s40520-018-1091-x

**Published:** 2018-12-17

**Authors:** Karin Werner, Anders Christensson, Helen Legrand, Mats Pihlsgård, Gunnar Sterner, Sölve Elmståhl

**Affiliations:** 1 Department of Geriatrics, Skåne University Hospital, Jan Waldenströms gata 35, 20502 Malmö, Sweden; 2 Department of Clinical Sciences Malmö, Lund University, Malmö, Sweden; 3 Department of Nephrology, Skåne University Hospital, Malmö, Sweden

**Keywords:** eGFR, CKD, RKFD, Mortality, Longitudinal, Older adults

## Abstract

**Background:**

The prevailing diagnostic criteria for CKD are age-independent, but have been challenged in light of the eGFR decline associated with normal aging. The stages of CKD communicate magnitude of risk of ESRD, cardiovascular morbidity, and mortality.

**Aims:**

This study aims to provide more insight into the morbidity and mortality associated with eGFR levels corresponding to the current CKD stages in older adults.

**Methods:**

The 2931 older adults in the Good Aging in Skåne study were randomized from the general population. The exposure variable used was eGFR level (CKD-EPI based on creatinine and cystatin C) with eGFR 60–89 mL/min/1.73 m^2^ as a reference; the outcomes were mortality, acute cardiovascular disease, congestive heart failure, and rapid kidney function decline (RKFD; defined as a decline in eGFR by 3 mL/min/1.73 m^2^ per year or more).

**Results:**

The mean age at baseline was 73 (SD 11) and mean follow-up time 11 (SD 5) years. Mortality was higher at lower eGFR levels with adjusted HR (95% CI) being 1.58 (1.34–1.88), 1.22 (1.05–1.41), 1 (reference), and 0.90 (0.67–1.21) for eGFR < 45, 45–59, 60–89 and ≥ 90 mL/min/1.73 m^2^, respectively. For acute CVD the adjusted HR (95% CI) were 1.23 (0.81–1.87), 1.21 (0.87–1.69), 1 (reference), and 0.53 (0.28–1.00) for the same eGFR levels.

**Conclusions:**

This study confirms that mortality in older adults increases with decreasing eGFR at eGFR levels below today’s threshold for CKD. The correlation was less certain for lower eGFR and incident cardiovascular disease.

**Electronic supplementary material:**

The online version of this article (10.1007/s40520-018-1091-x) contains supplementary material, which is available to authorized users.

## Introduction

The definition of chronic kidney disease (CKD) from the Kidney Disease: Improving Global Outcomes (KDIGO) guidelines puts eGFR at the center of the diagnosis. These guidelines were introduced in 2002 and have subsequently been revised, most recently in 2012 [1]. According to the KDIGO guidelines, even in the absence of other markers of kidney disease, a persistent eGFR below 60 mL/min/1.73 m^2^ is considered CKD. This threshold was originally chosen due to clinical experience and theoretical knowledge of kidney function. The rationale for the threshold has been strengthened by large studies demonstrating an increased risk of end-stage renal disease (ESRD), cardiovascular disease (CVD) and death when eGFR has dropped below 60 mL/min/1.73 m^2^ [2–4].

However, the usefulness of this cutoff point for older adults has come into question [5, 6], principally on the bases of the expected decline in organ function with age, the importance of competing risks with aging, the more benign progression of CKD in older adults, and a higher risk of adverse drug reactions in older adults from secondary prevention [5–10]. In addition, there is a stigma to being labeled with a chronic disease and care should be taken to develop medically meaningful disease criteria. Considering these factors, lowering the eGFR threshold for kidney disease to 45 mL/min/1.73 m^2^ in older adults without other signs of kidney damage has been proposed [5]. The defenders of today’s guidelines have however pointed to an increased relative risk of death and morbidity in older adults at eGFR levels below the current threshold of 60 mL/min/1.73 m^2^, though this risk is less pronounced than in younger people [2, 11].

The objective of this study is to explore the differences in outcomes between older individuals with eGFR levels equivalent to CKD stages 1, 2, 3a and 3b [12]. The outcomes in focus are well-known consequences of kidney disease, with mortality as the primary endpoint and incident acute CVD, incident congestive heart failure (CHF), ESRD and rapid kidney function decline (RKFD) as secondary endpoints. We also aim to describe the trajectory of eGFR in community-dwelling older adults. To improve the reliability of GFR estimation compared to earlier studies, we will use eGFR based on both creatinine (cr) and cystatin C (cys).

The overall aim of this study is to provide more insight on clinically relevant outcomes correlated to glomerular filtration rate in older adults. The results may be useful in future consideration of age-dependent diagnostic criteria for CKD.

## Methods

### Study design and setting

This prospective longitudinal study was conducted in Skåne, a region in southern Sweden. Study visits were at the study center or at home for those who had difficulties coming to the clinic.

Examinations were carried out at baseline, a 6-year follow-up, and a 12-year follow-up for all participants who were alive and consented. For those 78 years and older, additional examinations were carried out at 3 and 9 years after baseline (see flowchart in Appendix 2 for details). At each study visit the participants completed thorough questionnaires and underwent medical examinations including blood draws for laboratory measurements.

The baseline visit was carried out between 2001 and 2004. The last follow-up round ended in 2017. Of note is that the endpoints have different follow-up times. All-cause mortality was recorded from registries up until the end of the study in 2017. Information regarding incident acute CVD, CHF, and date of diagnosis was available from the national inpatient registry until the end of 2010.

### Participants

The participants of GÅS (Good Aging in Skåne) were randomized from the National Municipality Registry in five municipalities covering urban and rural areas in Skåne, a southern Swedish region. A total of 5370 men and women from nine age cohorts 60–93 years of age were invited by letter. Those who did not respond initially were invited again by letter and telephone. Invitees were considered non-eligible if they were deceased, had moved or were otherwise unreachable within 90 days of invitation.

The general exclusion criterion for the current study was the absence of laboratory measurements at baseline. There were also endpoint-specific exclusion criteria: for the endpoints acute CVD and CHF (congestive heart failure) all those with prior diagnoses of acute CVD or CHF at baseline were excluded. Where absolute and relative rate of decline were assessed at least two laboratory measurements were required.

### Variables (please see definitions in Appendix 1)

#### Exposure

The exposure tested was eGFR level at baseline: ≥ 90, 60–89 (reference), 45–59, or < 45 mL/min/1.73 m^2^ calculated by CKD-EPI_cr−cys_ based on both creatinine (cr) and cystatin C (cys). These categories are equivalent eGFR levels to CKD stages 1, 2, 3a, and 3b, respectively.

#### Outcome

The primary endpoint was death. The secondary outcomes were incident acute CVD (stroke and myocardial infarction), incident CHF (congestive heart failure), and RKFD defined as a decline in eGFR by 3 mL/min/1.73 m^2^ or more per year.

#### Predictors

In addition to the baseline eGFR level, we chose to study well-known cardiovascular risk factors as covariates: diabetes, treated hypertension, smoking, history of acute CVD, and history of CHF. The reason for including these risk factors is that they are known to influence both the exposure variable (eGFR) and the outcome variables (death, incident cardiovascular disease and kidney failure). For the outcomes of incident CVD and CHF we excluded those who had already experienced a cardiovascular event from the analysis.

### Statistical methods

All statistical analyses were performed using SPSS (Version 22.0. Armonk, NY, USA: IBM Corp.).

No statistical comparison of the descriptive data was performed [13].

Participants with incomplete data were not included in the model; the number of participants in each analysis is specified in the tables.

For mortality and acute CVD, Cox regression was used; for CHF and RKFD, logistic regression was used. For analysis with Cox regression and logistic regression the exposure variable (eGFR level) and the other covariates were simultaneously entered in the models. All variables relevant for analysis are described in Appendix 1 specifying data collection method and type of coding (categorical or continuous).

The absolute rate of change in eGFR was calculated by running linear regression analyses of eGFR against time separately for each participant and identifying the slopes. The relative rate of change was calculated as the absolute rate of change divided by the baseline eGFR. The total relative change in eGFR is the total change in eGFR divided by the baseline eGFR.

The Cox regression was conducted using the variables “Time to death/end of study” and “Time to incident acute CVD/death/end of study”. Thus, for the event incident acute CVD, the competing risk of death was taken into account by censoring on death. In model 1 for both outcomes there is only adjustment for sex and age. Model 2 is the fully adjusted model with additional covariates as specified in Table 2.

The logistic regression was performed using the binary logistic procedure controlling for covariates as specified in Table 3.

It is well known that when conducting Cox regression analyses there may be problems fulfilling the basic assumptions of proportional hazards. As a sensitivity analysis, we performed the Cox regression with a 5-year follow-up time (we expected that a shorter follow-up time would lead to smaller problems regarding fulfillment of model assumptions) and achieved similar results regarding the direction of effects as with the longer follow-up time.

A *p* value of ˂0.05 was considered statistically significant.

## Results

### Participants

The mean age (SD) of eligible non-responders was 77 (12) years compared to 73 (11) for participants at the moment of randomization. At follow-up visits non-participants were older than participants. The participation rate at baseline was 60% (2931/4893) (flowchart provided in Appendix 2). The mean follow-up time (SD) for the primary endpoint, mortality, was 11 (5) years and for the secondary outcomes incident acute CVD and CHF 6 (2) years, for RKFD 9 (3) years.

The proportion of participants older than 80 years increased with lower eGFR level: 0, 15, 61, and 84% at the eGFR levels ≥ 90, 60–89, 45–59, and ˂ 45 mL/min/71.73 m^2^, respectively. The baseline characteristics of study participants and the number missing data for each variable can be seen in Table 1.

**Table 1:** Descriptive characteristics of the baseline GÅS-population stratified by CKD stage (*n* = 2931)

	All	No eGFR at baseline	eGFR < 45 (CKD ≥ 3b)	eGFR 45–59 (CKD 3a)	eGFR 60–89 (CKD 2)	eGFR ≥ 90 (CKD 1)	Total missing
Categorical *n* (%)							
*N*	2931	116	442	586	1467	320	
*N* ≥ 80 years	989 (34)	51 (44)	369 (**83**)	355 (61)	214 (15)	0 (0)	0
Sex							0
Male	1295 (44)	41 (35)	162 (37)	229 (39)	665 (45)	198 (62)	
Female	1636 (56)	75 (65)	280 (63)	357 (61)	802 (55)	122 (38)	
Death	1472 (50)	72 (62)	413 (93)	439 (75)	496 (34)	52 (16)	0
Incident acute CVD	480 (16)	24 (21)	124 (28)	132 (23)	184 (13)	16 (5)	0
Incident CHF	404 (14)	23 (20)	151 (34)	116 (20)	109 (7)	5(2)	0
RKFD	178 (6)	0	17 (4)	38 (6)	91 (6)	32 (10)	0
Smoking							101
Current	474 (16)	14 (12)	27 (6)	68(12)	296 (20)	69 (22)	
Former	1067 (36)	34 (29)	153 (35)	193 (33)	545 (37)	142 (44)	
Never	1289 (44)	45 (39)	231 (52)	305 (52)	601 (41)	107 (33)	
Diabetes	229 (8)	12 (10)	54 (12)	48 (8)	89 (6)	26 (8)	16
History of acute CVD	431 (15)	29 (25)	136 (31)	105 (18)	143 (10)	18 (6)	15
History of CHF	516 (18)	33 (28)	177 (40)	145 (25)	144 (10)	17 (5)	12
Hypertension	849 (29)	45 (39)	208 (47)	227 (39)	315 (22)	54 (17)	26
Continuous mean (SD)							
Age (years)	73 (11)	75 (11)	85 (7)	80 (8)	69 (8)	63 (4)	0
Creatinine (µmol/L)	90 (28)	N/A	129 (45)	93 (16)	81 (14)	72 (11)	116
Cystatin C (mg/L)	1.17 (0.41)	N/A	1.88 (0.50)	1.06 (0.18)	0.92 (0.15)	0.81 (0.12)	116
eGFR_cr−cys_	66 (20)	N/A	35 (8)	53 (4)	74 (8)	97 (6)	116
Blood pressure (mmHg)	148/82 (24/11)	151/83 (23/13)	152/79 (28/13)	152/81 (24/11)	147/83 (23/11)	143/84 (21/11)	54

Percentages are column percentages and denote the percentage with the given trait within the categories specified in bold at the top row

eGFR was calculated by the CKD-EPI equations with the unit mL/min/1.73 m^2^ [28]

### Descriptive outcome data

Nearly half of the participants died during the follow-up period. There were 480 incident cases of acute CVD in total and 266 of these occurred in participants without prior diagnosis of MI, stroke, or CHF (*n* = 2103). There were 404 participants diagnosed with CHF and of these 186 did not have a history of MI, stroke or CHF at the baseline examination. There were only ten cases of ESRD diagnosed from baseline until the end of 2010. Since the incidence of ESRD was so small in this population, we chose not to use it in any detailed statistical analysis.

Because of the nature of the mortality and diagnosis registries available to us, there was no apparent loss to follow-up regarding mortality and incident CVD. However, for RKFD the loss to follow-up was substantial as 991 of the baseline participants had no follow-up laboratory measurement.

The mean rate of change in eGFR for the 1940 participants for whom we had access to at least two laboratory measurements was a decline of 0.9 (SD 1.7) mL/min/1.73 m^2^ per year. Relative to the baseline eGFR the average change was a yearly loss in eGFR of 1.4% over the course of the study period. One hundred eighty-six individuals had a total decrease in eGFR by 40% or more over a mean follow-up period of 9 years ranging from 2 to 15 years. There were 178 cases of RKFD. Details of changes in eGFR for the whole group and subgroups, defined according to the eGFR level at baseline, can be seen in Appendix 3.

### Association between CKD stage and mortality

A lower eGFR level was associated with an increased mortality (Table 2). This pattern did not change when the Cox model was fully adjusted. Compared to the reference eGFR level 60–89 mL/min/1.73 m^2^ there was a 22% increased risk of death for those with eGFR between 45 and 60 mL/min/1.73 m^2^. Those with the lowest eGFR had an even more pronounced risk with a 58% increase in mortality compared to the reference group. See Table 2 for complete results and confidence intervals.

**Table 2:** Baseline eGFR category and hazard ratios for all-cause mortality and incident acute cardiovascular disease in the GÅS-population

	Mortality model 1	Mortality model 2	CVD model 1	CVD model 2
eGFR level				
≥90 (CKD 1)	**0.85 (0.63–1.13)**	0.90 (0.67–1.21)	**0.49 (0.26–0.93)**	0.53 (0.28–1.00)
60–89 (CKD 2)	**1 (ref)**	1 (ref)	**1 (ref)**	1 (ref)
45–59 (CKD 3a)	**1.26 (1.09–1.46)**	1.22 (1.05–1.41)	**1.30 (0.93–1.80)**	1.21 (0.87–1.69)
<45 (CKD ≥ 3b)	**1.79 (1.52–2.10)**	1.58 (1.34–1.88)	**1.54 (1.03–2.31)**	1.23 (0.81–1.87)
Age, per 1 year increase	**1.11 (1.10–1.12)**	1.12 (1.11–1.13)	**1.06 (1.04–1.08)**	1.07 (1.05–1.09)
Female	**0.64 (0.57–0.71)**	0.67 (0.59–0.76)	**0.67 (0.52–0.86)**	0.73 (0.56–0.95)
Smoking status				
Current		1.98 (1.67–2.35)		2.00 (1.39–2.88)
Former		1.13 (0.99–1.28)		1.37 (1.01–1.84)
Never		1 (ref)		1 (ref)
Diabetes		1.52 (1.27–1.82)		2.71 (1.85–3.97)
History of acute CVD		1.28 (1.12–1.48)		N/A
History of CHF		1.34 (1.18–1.53)		N/A
Treated for hypertension		1.12 (1.00–1.26)		1.50 (1.14–1.97)

Numbers provided are hazard ratios (95% CI). EGFR was calculated by the CKD-EPI equation based on cystatin C and creatinine with the unit mL/min/1.73 m^2^ [28]

Model 1 (bold text) is adjusted only for age and sex

Model 2 is adjusted for age, sex, smoking, diabetes, and treated hypertension. For mortality model 2 is also adjusted for prior MI, stroke, and prior CHF

For mortality model 1 all participants with laboratory tests at baseline (*n* = 2815) were included. For mortality model 2 all participants with complete data were included (*n* = 2718). Study period is from the baseline examination (2001–2004) until study closure 2017-05-16 or date of death

For CVD model 1 all participants with laboratory measurements and without a prior diagnosis of acute CVD or congestive heart failure at baseline (*n* = 2103) were included. For CVD model 2 all participants from model 1 with complete data were included (*n* = 2059). The study period ran from the baseline examination up until 2010-12-31 or date of death if no event occurred

### Association between CKD stage and incident cardiovascular disease and RFKD

Generally, the relative hazards for the endpoint incident acute CVD (MI/stroke) were not statistically significant but were of the same magnitude and of similar pattern as for mortality.

Given the typically inexact timing of onset of CHF and RFKD, these endpoints were investigated with logistic regression; see Table 3 for details. The risk pattern for the four levels of eGFR and the outcome heart failure resembles that found for death and acute CVD in the Cox analysis. The risk of CHF was lower for those with higher eGFR and higher for those with low eGFR compared to the reference group with eGFR 60–89 mL/min/1.73 m^2^. The odds for CKD stage 3a were not significantly elevated compared to the reference.

**Table 3:** Baseline eGFR category and odds ratios for incident congestive heart failure and rapid kidney function decline (RKFD) in the GÅS-population

	CHF model 1	CHF model 2	RKFD model 1	RKFD model 2
eGFR level				
≥90 (CKD 1)	**0.30 (0.09–0.96)**	0.33 (0.10–1.06)	**3.22(1.99–5.23)**	3.29 (2.01–5.38)
60–89 (CKD 2)	**1 (ref)**	1 (ref)	**1 (ref)**	1 (ref)
45–59 (CKD 3a)	**1.15 (0.74–1.80)**	1.01 (0.64–1.60)	**0.57 (0.36–0.89)**	0.48 (0.30–0.77)
<45 (CKD ≥ 3b)	**2.27 (1.40–3.70)**	1.69 (1.01–2.83)	**0.32 (0.17–0.59)**	0.25 (0.13–0.47)
Age. per 1 year increase	**1.08 (1.06–1.10)**	1.09 (1.07–1.12)	**1.09 (1.07–1.11)**	1.09 (1.07–1.17)
Female	**0.62 (0.44–0.86)**	0.57 (0.40–0.82)	**0.89 (0.64–1.23)**	0.89 (0.63–1.26)
Smoking status				
Current		1.51 (0.91–2.51)		1.44 (0.90–2.31)
Former		1.13 (0.76–1.69)		0.90 (0.62–1.31)
Never		1 (ref)		1 (ref)
Diabetes		1.21 (0.65–2.26)		1.48 (0.84–2.61)
History of acute CVD				1.08 (0.68–1.73 m)
History of CHF				1.49 (0.96–2.32)
Treated for hypertension		1.96 (1.37–2.81)		1.66 (1.16–2.37)

Numbers provided are odds ratios (95% CI). EGFR was calculated by the CKD-EPI equation based on cystatin C and creatinine with the unit mL/min/1.73 m^2^ [28]

Model 1 (bold text) is adjusted for age and sex

Model 2 is adjusted for age, sex, smoking, diabetes, and treated hypertension. For RFKD model 2 is also adjusted for prior acute CVD and prior CHF

For CHF model 1 all participants with laboratory measurements and without a prior diagnosis of acute CVD or congestive heart failure at baseline (*n* = 2103) were included. For CHF model 2 all participants in model 1 with complete data were included (*n* = 2059). The study period ran from the baseline examination up until 2010-12-31 or until death

For RFKD model 1 and 2 all participants with eGFR at baseline and one or more additional eGFR (*n* = 1940) were included. A rate of decline of 3 mL/min/1.73 m^2^ or more per year were considered RFKD. The study period for each participant ran from the baseline examination (2001–2004) until their last laboratory measurement (2005–2017)

The risk of rapid decline in eGFR was higher for those who started out at a higher baseline eGFR level. Older age was also associated with higher odds of RKFD.

## Discussion

This study has explored the relationship between eGFR in older adults and important consequences of renal function during an up to 16-year-long follow-up period. As expected the level of eGFR and rate of mortality showed an inverse relationship; lower levels of eGFR correspond to a higher mortality rate starting at the eGFR level below 60 mL/min/1.73 m^2^ which is pathological by the criteria of today. A similar but not uniformly statistically significant tendency was seen for the risk of acute CVD and occurrence of CHF. The mean rate of change in eGFR was a decrease of almost 1 mL/min/1.73 m^2^ per year corresponding well to the generally accepted age-related rate of decline [14–16].

There are now several studies exploring the relationship between level of eGFR and subsequent risk of mortality and morbidity in older adults [2, 17–19]. They all report an association between mortality and lower levels of kidney function, but there are discrepancies regarding the eGFR point of risk increase, the risk amplitude, and whether the association is U-shaped. One reason for conflicting results can be attributed to the limitations of creatinine as a marker for eGFR. This is highlighted by findings from the older general population cohort “Health and Anemia”, in which different eGFR equations yielded very different prevalences of CKD and demonstrated a varying capacity to predict mortality [20]. The inclusion of cystatin C in estimating equations has been shown to improve GFR estimation [21] in our population of older adults. Some previous studies have shown improved prediction of mortality with the use of cystatin C in eGFR equations [18, 22, 23], with the exception of the recent results from the InChianti study population, in which the addition of cystatin C did not show a difference in mortality prediction compared to creatinine-only equations [24].

To our knowledge, our study is the first in older adults from the general population that has used a validated two-marker eGFR looking at mortality and morbidity as endpoints.

The present study is smaller than Hallan’s multicenter study [2], but the monocentric design is a strength. The results from the aforementioned study cannot be directly compared with the present results as the previous study used a different reference eGFR level of 80 mL/min/1.73 m^2^ and creatinine-based eGFR. Still, the results from the two studies are consistent. Looking at all-cause mortality and eGFR < 45 mL/min/1.73 m^2^, the earlier study found point hazard ratios of 1.35–1.59 in older age groups, compared to 1.58–1.79 in our study.

A Swedish cohort study used creatinine-based eGFR level and a shorter follow-up in the survival analysis and found much more elevated hazard ratios for all-cause mortality at CKD stage 3b than we did [17]. We cannot find a single obvious explanation for the difference in magnitude of the estimated effect but the studies differ in several important ways: their participants were all women aged 75 years at baseline, the choice of reference level for eGFR is slightly different as our reference category excludes those with highest eGFR (more than 90 mL/min/1.73 m^2^), and the number of participants with CKD stage ≥ 3b was only 27 compared to 442 in our study.

Somewhat surprisingly, the secondary outcome incident acute CVD had only a modest association with eGFR level and the association weakened in the fully adjusted model rendering possible a distinction between the direct effect of eGFR and the total effect of eGFR and vascular risk factors. Of course the increased mortality hazard with lower eGFR most probably reflects death due to cardiovascular disease.

A higher eGFR level was generally associated with better outcomes, but for RKFD the relationship was reversed. Part of this can perhaps be explained by the choice of an absolute measure instead of a relative measure of decline. Moreover, it could be caused by the imprecision of eGFR equations at higher eGFR levels. The group with the lowest eGFR level had relatively few cases of RKFD and it should be noted that more than half did not come to a second study visit with laboratory measurements because they were sicker, older and died at a higher rate. Comparisons to earlier studies are not easy due to important methodological differences. Still, we consider the proportion of participants with RKFD low compared to a US Veteran’s Affairs study where the proportion of older adults with RKFD was more than double compared to our study [8]. They also found that older adults experience lower odds of absolute RKFD compared to younger adults at the same eGFR level [8]. This and our finding of a remarkably low level of RFKD in older adults with low eGFR could be explained by the different pathophysiology behind an equivalent eGFR in a younger person, depending on the proportion of disease- versus age-related decline.

There are limitations to our study that may influence the interpretation of the results. The study population is randomized from the general older population. Due to selection bias we suspect that the study sample is healthier than the general age-matched population of Skåne. Pathophysiological associations should normally be robust for this [25]. Therefore, possible selection bias should not invalidate the conclusions of this study. The attrition when it comes to follow-up visits and laboratory measurements is more problematic and could affect the measures of change in eGFR and thus the estimated incidence of RKFD. Only a third of the original cohort made it to the last assessment about 15 years after baseline, typically due to death since nearly half of the participants passed away. We also expect the sickest not to come to the follow-up visits. To counteract this tendency, home visits were offered.

Furthermore, there is possible misclassification in the exposure variable eGFR. There are general and study-specific uncertainties in its use as a surrogate for GFR and kidney function. In the present cohort we found that 19% of the participants have more than 30% difference between eGFR_cr_ and eGFR_cys_ at baseline [26]. In most cases eGFR_cr−cys_ is more reliable than eGFR based on either marker alone. This has been validated in several studies of older adults [27], including a subgroup of the GÅS population [21]. The eGFR used throughout this study was thus calculated by the CKD-EPI_cr−cys_ [28]. Moreover, there were changes in the creatinine and cystatin C laboratory methods between the follow-up visits which is not unique to the present study [29]. For the classification of chronic kidney disease there must be repeated measures of the GFR markers at least 3 months apart. We only had one measurement point at each study visit increasing the likelihood of a false positive diagnosis of CKD, which is why we refer to eGFR level rather than CKD stage [6]. In addition, no urine samples were collected, which is important for CKD staging and risk assessment. This is especially problematic as proteinuria has been suggested to be of major importance especially in older adults in order to differentiate age-related decline in eGFR from chronic kidney disease [5].

The group with eGFR above 90 mL/min/1.73 m^2^ might be heterogeneous and include those with kidney disease causing hyperfiltration. There may also be misclassification error in other covariates: diabetes was self-reported or retrieved from hospital and primary care records, and treated hypertension was used as a proxy for hypertension.

In contrast, there is no important risk of misclassification for the outcomes of death, stroke, myocardial infarction, and ESRD. The diagnosis and time of onset for CHF can be more ambiguous. Although the registries employed to gather CHF data have excellent coverage, only diagnoses made on inpatient wards are included, which increases the likelihood of false negatives. The choice of measure for RKFD was not obvious, but due to the nature of the data we had access to, with few measurement points at long intervals and a long follow-up period, an absolute decline of 3 mL/min/1.73 m^2^ per year seemed most appropriate. This measure of rapid decline has been used in previous high quality studies [30, 31]. A harder endpoint for kidney disease progression would naturally be ESRD, but there were few cases among our participants.

In a study of older adults age is a dominant risk factor for almost any outcome. It is therefore a challenge to account for this in an appropriate manner. Of note in this study is that the exposure groups are very different in composition, especially when it comes to age: individuals over 80 years of age made up a majority of the group with eGFR below 45 mL/min/1.73 m^2^ and were completely absent in the group with eGFR above 90 mL/min/1.73 m^2^. **F**inding the best available analysis method can be challenging in older individuals. Risk models may have difficulties as there is a natural limit to survival despite the magnitude of other effects on the outcome.

Despite these limitations, the results of this study have contributed to our knowledge on the effect of eGFR level on mortality and cardiovascular and renal morbidity. It is safe to say that an eGFR below the current threshold for CKD is a risk factor for mortality and possibly cardiovascular disease in older adults, but given the magnitude of the risk this may not automatically justify a disease label. The CKD category 3a includes patients with mostly age-related structural and functional changes in the kidney as well as patients with renal disease often as a consequence of age-related cardiovascular risk factors and disease Importantly, CKD follow-up and treatment decisions for older adults should not automatically be the same as for younger adults with higher relative risks, less ambiguous treatment results, and longer life expectancies [2, 10, 32, 33].

In conclusion, this study confirms that mortality in older adults increases with decreasing eGFR at GFR levels below today’s threshold for CKD of 60 mL/min/1.73 m^2^. Whether the magnitude of the increased risks associated with a lower eGFR in older adults are sufficient for keeping the same diagnostic thresholds for CKD as in younger adults is still up for discussion. Future studies could focus on other outcomes that may be even more important for an older individual, such as activities of daily living and quality of life.

## Electronic supplementary material

Below is the link to the electronic supplementary material.

Supplementary material 1 (DOCX 27 KB)

Supplementary material 2 (PDF 259 KB)

Supplementary material 3 (DOCX 12 KB)
