# Supplementary material for: Cystatin C and creatinine-based eGFR levels and their correlation to long-term morbidity and mortality in older adults
Source: Aging Clin Exp Res. 2018 Dec 17;31(10):1461–9. doi: 10.1007/s40520-018-1091-x (PMC6763515; doi:10.1007/s40520-018-1091-x)
Supplement: Supplementary file 1 — Supplementary material 1 (DOCX 27 KB) [file 40520_2018_1091_MOESM1_ESM.docx]

**Appendix 1**

**Detailed collection methods and coding for key variables presented in “Cystatin C and creatinine based eGFR levels and their correlation to morbidity and mortality in older adults”**

| **Variable** | **Collection method** | **Coding** |
| --- | --- | --- |
| Sex | Extracted from personal identity number, which includes coding for sex | Male or female |
| Age at baseline | Calculated from study visit date (when baseline variables were collected) and personal identity number, which includes birth date | Continuous variable |
| Smoking | Self-reported by participants in a questionnaire; possible answers to the question “do you smoke” were: “yes, regularly,” “yes, occasionally,” “no, no longer smoke,” and “no, have never smoked.” | Three categories: 1.Regular or occasional smoking classified as current, 2. No longer smoker classified as former, 3. Never smoked |
| Hypertension | Medical questionnaire completed by physician based on the medical record and participant report. Question was “is patient currently under treatment for hypertension.” | Yes or no |
| Diabetes | Medical questionnaire completed by physician based on the medical record and participant report. One question for diabetes type 1 and one question for diabetes type 2 were fused into one variable “Diabetes” | Yes or no |
| Creatinine | Baseline: Plasma stored in a biobank until analysis in 2007, analyzed by Jaffé method on Beckman UniCel DxC800 with reagent from Beckman (CREm). CV at 70 µmol/L 8 %, at 360 µmol/L 5 % and at 660 µmol/L  3 %. Calibrator SYNCHRON® Systems AQUA CAL 1 and 2, correlated to an IDMS method.  Follow-up: Y 3, Y 6, Y 9: Analyzed in 2014 from frozen plasma. Creatinine was analyzed by the enzymatic colorimetric method on Cobas 8000 with reagent (Creatinine plus version 2) and calibrator  (f.a.s.) from Roche. CV at 70 µmol/L 1,4% and CV at 600 µmol/L 1,7%. Calibrated to values assigned by IDMS and traceable to SRM 914a.  Follow up Y 12: Analyzed as above but from fresh plasma. | Continuous, used to calculate eGFR |
| Cystatin C | Baseline: Plasma stored in a biobank until analysis in 2007. Analyzed by a particle-enhanced immunoturbidimetric assay on biobank plasma analyzed in 2007, with reagent Gentian on Beckman Synchron LX20. CV at plasma cystatin C concentration of 1, 3.48, and 6 mg/L were 3.3, 0.9, and 1.0 % respectively.  Follow-up: Y 3, Y 6, Y 9: Analyzed in 2014 (September 14-January 15) from frozen plasma. Analyzed by a particle-enhanced immunoturbidimetric assay on Cobas 8000. Reagent (Cystatin C Gen 2) and calibrator (f.a.s.) were from the second generation (Roche 2). The CV for Roche 2 was 2.2 and 1.1% at concentrations of 0.6 and 2.0 mg/L, respectively. The calibration is traceable to the certified reference material ERM-DA471/IFCC.  Follow up Y12: Analyzed as above but from fresh plasma from June 2014 (n=765). Earlier analysis (n=431) was analyzed on the same instrument but with the first generation Roche (Roche 1), reagent (f.a.s.) with CV 4,8 and 3.7% at concentrations of 1,0 and 4,2 mg/L, respectively. | Continuous, used to calculate eGFR |
| eGFR | Calculated by the combined CKD-EPI equation[1] based on both creatinine and cystatin C which has been validated in the study population[2] (Unit: mL/min/1.73m^2^)$135 \times min((cr/88.4)/\kappa, 1)\alpha\times max((cr/88.4)/\kappa, 1)-0.601\times min(cys/0.8, 1)-0.375 \times max(cys/0.8, 1)-0.711\times0.995\mathrm{Age} [\times0.969 if female] [\times1.08 if black]$  κ is 0.7 for females and 0.9 for males.  α is -0.248 for females and -0.207 for males, min indicates the minimum of ($cr/88.4)/\kappa$ or 1, and max indicates the maximum of (cr/88.4)/κ or 1. | Continuous |
| CKD stages | Comparison between reference CKD stage 2 (eGFR ≥60, <90 mL/min/1.73m^2^) and CKD-stage 1 (eGFR≥90 mL/min/1.73m^2^), CKD stage 3a (eGFR ≥45, <60 mL/min/1.73m^2^), CKD stage 3b-5 (eGFR <45 mL/min/1.73m^2^) | 4 categories: CKD stage 1, 2, 3a, 3b-5 |
| History of CVD | MI, stroke (all types) recorded by research doctor with information from hospital records and patient history at baseline visit | Yes or no |
| History of CHF | Congestive heart failure NYHA 1-4 recorded by research doctor with information from hospital records and patient history at baseline visit | Yes or no |
| Date of death | Date of death was drawn from the regional municipality registry which draws data from The Swedish Tax Agency that receives data from the mandatory death certificates issued by a doctor. | Date |
| Time to death/end of study | Time (years) elapsed between study entry and date of death or end of study May 2017. | Continuous |
| Censoring | Time elapsed between study entry (individual) and study closure on May 15^th^ 2017 (no drop-out). | Continuous |
| Incident acute CVD | Myocardial infarction ICD 10 code I21 or stroke (I61, I63, I64). Information retrieved after baseline from the Swedish inpatient registry from study entry up until December 31, 2010 | Yes or no |
| Time to incident acute CVD/death | Time (years) elapsed between study entry and first occurrence of an incident acute CVD, death, or censoring on December 2010. | Continuous |
| Incident chronic heart failure | Occurrence of the ICD 10 diagnosis congestive heart failure (I50, I11.0, I13.9, I13.2) after baseline. Information retrieved from the Swedish inpatient registry from study entry up until December 31, 2010 | Yes or no |
| Incident ESRD | Occurrence of an ICD 10 diagnosis coded N186, N185, Z99.2, and/or Z940 in the Swedish inpatient registry from study entry up until December 31, 2010 | Yes or no |
| ΔeGFR per year | Change in eGFR per year from individual regression line derived from all available laboratory measurements divided by baseline eGFR | Continuous |
| ΔeGFR per year relative to baseline eGFR | $\Delta eGFR per year relative to baseline eGFR$ | Continuous |
| RKFD | ≥3 mL/min/1.73m^2^ per year decline in eGFR over the study period. Rate of decline determined by linear regression from the GÅS laboratory measurements. | Yes or no |

1. Inker LA, Schmid CH, Tighiouart H, Eckfeldt JH, Feldman HI, Greene T, Kusek JW, Manzi J, Van Lente F, Zhang YL, Coresh J, Levey AS, Investigators C-E (2012) Estimating glomerular filtration rate from serum creatinine and cystatin C. The New England journal of medicine 367 (1):20-29. doi:10.1056/NEJMoa1114248

2. Werner K, Pihlsgard M, Elmstahl S, Legrand H, Nyman U, Christensson A (2017) Combining Cystatin C and Creatinine Yields a Reliable Glomerular Filtration Rate Estimation in Older Adults in Contrast to beta-Trace Protein and beta2-Microglobulin. Nephron 137 (1):29-37. doi:10.1159/000473703
