# Supplementary material for: Cystatin C and creatinine-based eGFR levels and their correlation to long-term morbidity and mortality in older adults
Source: Aging Clin Exp Res. 2018 Dec 17;31(10):1461–9. doi: 10.1007/s40520-018-1091-x (PMC6763515; doi:10.1007/s40520-018-1091-x)
Supplement: Supplementary file 2 — Supplementary material 2 (PDF 259 KB) [file 40520_2018_1091_MOESM2_ESM.pdf]

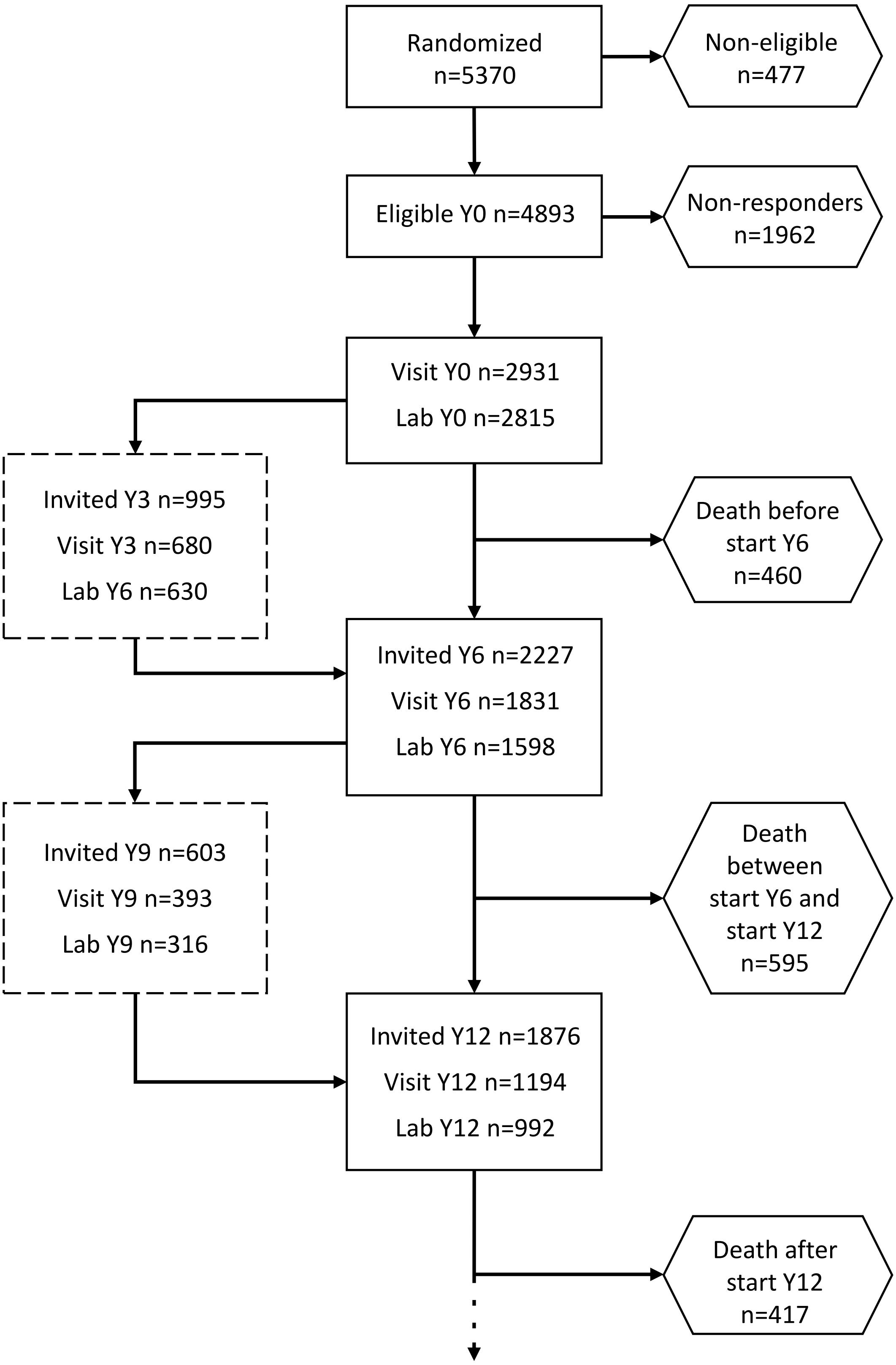

Figure 1. Flowchart for the GÅS baseline cohort for the visits year (Y) 0, 3, 6, 9, and 12

Note dotted lines around study visits Y3 and Y9, which only involve participants older than 78 years of age.
