# Supplementary material for: Cystatin C and creatinine-based eGFR levels and their correlation to long-term morbidity and mortality in older adults
Source: Aging Clin Exp Res. 2018 Dec 17;31(10):1461–9. doi: 10.1007/s40520-018-1091-x (PMC6763515; doi:10.1007/s40520-018-1091-x)
Supplement: Supplementary file 3 — Supplementary material 3 (DOCX 12 KB) [file 40520_2018_1091_MOESM3_ESM.docx]

**Appendix 3**

**Descriptive presentation of rate of change in eGFR for the GÅS population* (n=1940)**

| **Participants** | **All** | **eGFR <45 (CKD ≥3b)** | **eGFR 45-59 (CKD 3a)** | **eGFR 60-89 (CKD 2)** | **eGFR≥90**  **(CKD 1)** |
| --- | --- | --- | --- | --- | --- |
| N* (% of participants with baseline eGFR) | 1940 (69) | 196 (44) | 364 (62) | 1132 (77) | 248 (78) |
| Mean ΔeGFR per year | -0.9 (1.7) | -0.6 (1.8) | -0.9 (1.9) | -0.9 (1.7) | -1.4 (1.1) |
| Mean percentage ΔeGFR per year relative to baseline eGFR | -1.4 (3.0) | -1.7 (5.3) | -1.8 (3.6) | -1.2 (2.3) | -1.4 (1.6) |
| N with decline in eGFR ≥3 mL/min/1.73m^2^ per year | 178 (9) | 17 (9) | 38 (10) | 91 (8) | 32 (13) |
| N with eGFR decline ≥40% over study period | 186 (10) | 27 (14) | 44 (12) | 95 (8) | 20 (8) |

*All participants at the baseline GÅS examination with eGFR at baseline and at least one more eGFR during the study period.

Numbers provided are n (%) and mean (SD). EGFR was calculated by the CKD-EPI equation based on cystatin C and creatinine with the unit mL/min/1.73m^2^
